# PSG7 indicates that age at diagnosis is associated with papillary thyroid carcinoma: A study based on the cancer genome atlas data

**DOI:** 10.3389/fgene.2022.952981

**Published:** 2022-10-05

**Authors:** Tianjie Tian, Zixiong Zhang, Ting Chen

**Affiliations:** ^1^ Shengli Clinical Medical College of Fujian Medical University, Fuzhou, China; ^2^ Department of Otorhinolaryngology Head and Neck Surgery, The Central Hospital of Enshi Tujia and Miao Autonomous Prefecture, Enshi, China

**Keywords:** PSG7, papillary thyroid carcinoma, age, prognosis, biomarker, TCGA, GEO

## Abstract

The age of the patients at diagnosis (age at diagnosis) is a self-contained element of danger for the prognosis of patients with papillary thyroid carcinoma (PTC), which has been well recognized and continuously adopted by the international cancer staging system. However, few studies have investigated its intrinsic mechanisms. In this study, we aim to comprehensively reveal the age-related pathogenesis of PTC and identify potential prognostic biomarkers. We divided the samples into two groups, young and elderly, to filter differentially expressed genes in The Cancer Genome Atlas (TCGA), with an age of 55 years serving as a cutoff. Moreover, we combined univariate, LASSO, and multivariate Cox regression analyses to construct age-related signatures for predicting progression-free survival. Additionally, functional enrichment analysis, immune infiltration analysis, differential expression analysis, clinicopathological correlation analysis, and drug sensitivity analysis were performed in different risk subgroups and expression subgroups. We screened 88 upregulated genes and 58 downregulated genes. Both the LASSO regression model that is validated in TCGA and the model of six age-related prognostic genes (IGF2BP1, GPRC6A, IL37, CRCT1, SEMG1, and PSG7) can be used to evaluate the progression-free survival of PTC patients. The GO, KEGG, and GSEA analyses revealed that each key gene was closely associated with PTC development. Furthermore, CD8^+^ T cells decreased significantly, while regulatory T cells increased dramatically in the high-risk and PSG7 high expression groups. PSG7 was remarkably correlated with clinicopathological parameters (pathologic stage, T stage, and N stage) of PTC patients, and PSG7 expression was elevated in tumor samples from both TCGA and the Gene Expression Omnibus and was strongly associated with progressive stage and poor prognosis. Our results provide an innovative understanding of the age-related molecular mechanisms of PTC development. PSG7 was identified to exert a critical role in PTC progression and may serve as a promising strategy for predicting the prognosis of PTC.

## 1 Introduction

Over the last 30 years, the prevalence of thyroid carcinomas has been increasing rapidly by 6.2% each year, which is partly attributed to over-screening, and ranks fifth among all female malignancies and first for women under 25 years of age (Cramer et al., 2010; Bao et al., 2021). Papillary thyroid carcinoma (PTC) is the most frequent histopathological subtype, representing nearly 90% of all thyroid carcinomas, and its incidence has been increasing, while the incidence of other forms has remained unchanged (Miranda-Filho et al., 2021; Rossi et al., 2021). Patients with PTC who undergo a series of formal personalized therapies, including surgery, iodine-131 treatment, and suppression treatment with exogenous hormones have an excellent prognosis with a five-year disease-specific survival rate > 98% (Schmidbauer et al., 2017; Wang and Sosa, 2018). However, when the age at diagnosis exceeds a certain threshold, the overall prognosis worsens because of the increased cause-specific mortality in patients with PTC (Londero et al., 2015), and the mechanisms underlying this phenomenon remain elusive. To uncover this mechanism further, the abundant microarray and sequencing data available in public databases combined with advanced computational and bioinformatics approaches indicate a novel direction for our study; to improve the clinical outcomes of this subset of patients, targeted therapy and immunotherapy should become an attractive alternative therapeutic intervention in refractory PTC (Al-Jundi et al., 2020; Sun et al., 2021). Thus, a deeper exploration of the underlying mechanisms and therapeutic targets for PTC is of fundamental importance to facilitate individualized therapeutic approaches.

Recently, proto-oncogene B-Raf (BRAF), rat sarcoma (RAS), and telomerase reverse transcriptase (TERT) promoter mutations have been shown to strongly precede the development of PTC (Tanaka et al., 2019; Al-Salam et al., 2020; Traversi et al., 2021). Additionally, rearrangements, including rearranged during transfection (RET) and neurotrophic tyrosine kinase receptor type 1 (NTRK1), can accelerate the progression of the malignancy by activating mitogen-activated protein kinase signaling (Pekova et al., 2020; Salvatore et al., 2021). Moreover, non-coding RNA regions can also be implicated in the tumorigenesis of PTC in various forms, including microRNAs, lncRNAs, and circRNA (Gou et al., 2018; Al-Abdallah et al., 2020; Wu et al., 2020). Most intriguingly, the risk of PTC in carriers of the rs944289 single nucleotide polymorphism (SNP) was increased per year of age (Kula et al., 2017); there were significantly more TERT mutations in PTC patients ≥ 55-years-old (Park et al., 2021), which portended a poor prognosis likely by promoting immortalization and genomic instability in two phases (Chiba et al., 2017). Although numerous studies have been conducted, the detailed mechanisms underlying PTC carcinogenesis remain elusive.

Although the personalized strategy of diagnosis and treatment based on specific biomarkers can facilitate the evaluation of risk stratification, guide therapy, and evaluate prognosis, its practicality is insubstantial, owing to a skewed cost–benefit analysis and current limitations for clinical application (Subbiah et al., 2018; Norris et al., 2020). Thus, we appear to benefit the most from the combination of microscopic biomarkers and macroscopic clinical information by elucidating the associated mechanisms between them. Surprisingly, according to the major constituents of tumor depth (T), locoregional lymphatic node status (N), and distant metastasis (M), the American Joint Committee on Cancer (AJCC) thyroid cancer staging system has played the most significant role worldwide in providing prognostic information and evaluating the prognosis of thyroid cancer. It is widely acknowledged that age at diagnosis is a self-contained element of danger for the outcome of PTC patients (Kim et al., 2017; Kauffmann et al., 2018). The cutoff value of age at diagnosis increased from 45 to 55 years, which is one of the most prominent features of the AJCC 8th staging system for judging the prognosis of individuals with differentiated thyroid carcinoma, allowing surgeons to better stratify patients to avoid radical surgical procedures and postoperative treatments (Amin et al., 2017; Thewjitcharoen et al., 2021). Furthermore, one study combined clinical data with genetic data (differential gene expression, copy number variation, classical pathway alteration, and somatic mutation) and discovered that this current version for thyroid cancer can predict the recurrence rate and survival more accurately compared with the previous version (Kim et al., 2018). However, the investigators did not further establish an age-related model for validation and performed a more in-depth molecular mechanistic study. In addition, without considering specific molecular markers, age at diagnosis alone cannot be an ideal guideline for survival prognosis judgment, precise risk stratification, or scientific guidance for treatment decision-making. Therefore, a novel strategy to determine the precise mechanism of PTC progression with age will provide remarkable benefits for individualized cancer care.

In the present study, we aim to elucidate the underlying mechanisms by which an increase in age can affect the prognosis of patients with PTC, identify the specific prognosis biomarkers for PTC, and analyze the relationship between the expression levels of the key genes and drug sensitivity by bioinformatics analyses. PTC datasets from The Cancer Genome Atlas (TCGA) and Gene Expression Omnibus (GEO) databases will be used for differential expression analysis, model construction, enrichment analysis, survival analysis, clinicopathological correlation analysis, immune infiltration analysis, and drug sensitivity analysis. Among the meaningful results, one of the most prominent findings was that PSG7 was first identified as the most promising age-related prognostic biomarker, from which we gained valuable insight into prognostic evaluation and therapeutic guidance for individuals with PTC.

## 2 Materials and methods

### 2.1 Data acquisition and preprocessing

We obtained thyroid carcinoma datasets [The Cancer Genome Atlas Thyroid Cancer (TCGA-THCA; *n* = 553 cases)] from the TCGA database using the TCGAbiolinks R package (Colaprico et al., 2016). A total of 545 samples from patients with PTC and healthy individuals were included in the analysis after removing eight cases of non-PTC. The matched clinical information of the THCA-TCGA dataset, including patient sex, survival status, follow-up time, pathological stage, and other details, was obtained after converting the selected data types of counts and FPKM to TPM.

We also obtained thyroid cancer datasets from the GEO database using the GEOquery package (Davis and Meltzer, 2007). The GSE29265 dataset, which was from the species Homo sapiens and based on the GPL570 platform, consisted of 20 samples with PTC, nine samples with undifferentiated thyroid carcinoma, and 20 healthy samples. Using the R package limma (Ritchie et al., 2015) to standardize the data of the two groups, 20 PTC samples and 20 healthy samples were enrolled in this study (Supplementary Table S1).

### 2.2 Identification of differentially expressed genes

Growing lines of studies and the latest American Joint Committee on Cancer staging system for differentiated thyroid carcinoma have indicated that the cut-off value of age at diagnosis used for staging increasing from 45 to 55 can allow low-risk patients to avoid aggressive surgical resections and postoperative therapies, improve disease management strategies, and reduce psychosocial and financial burdens (Mazurat et al., 2013; Amin et al., 2017; Pontius et al., 2017; Kim et al., 2018; Shteinshnaider et al., 2018; Thewjitcharoen et al., 2021). That is, when the age at diagnosis exceeds 55, the overall prognosis worsens because of the increased cause-specific mortality in patients with PTC, and the mechanisms underlying this phenomenon remain elusive. In order to seek the age-related genes and explore the underlying molecular mechanisms, we separated the PTC samples from the TCGA database into a young group (*n* = 344) and an elderly group (*n* = 145), with the age of 55 years serving as a cutoff. We then analyzed the differentially expressed genes (DEGs) using the DESeq2 R package (Love et al., 2014), with a significance threshold set at an adjusted *p* value < 0.05. Genes with Log2FC value ≥ 1 were considered to be upregulated and genes with Log2FC value ≤ 1 were downregulated in the disease group, which were designated as age-related genes.

Subsequently, risk scores were calculated for all samples using a multivariate Cox regression model for further analysis. Immediately thereafter, they were split into low- and high-risk groups using the median value of the risk scores. Similarly, we performed DEGs analysis using the DESeq2 R package (Love et al., 2014), with a significance threshold set at an adjusted *p* value < 0.05. Genes with Log2FC value ≥ 1 were deemed to be upregulated and genes with Log2FC value ≤ 1 were deemed to be downregulated in the disease group, which was designated as an age-related gene.

### 2.3 Construction of protein-protein interaction networks

The STRING database (Szklarczyk et al., 2017) is adept in searching for interactions between the known and predicted proteins, which includes 2031 species, information on 9.6 million proteins, and 13.8 million protein interactions. Specifically, as an extremely helpful database, it is enriched in data containing the experimental outcomes, information obtained by text mining of PubMed abstracts, integrated data from other databases and predictive bioinformatics data. In our study, it was utilized for constructing the protein–protein interaction (PPI) network of DEGs between the young and elderly groups with a confidence score (0.400); the PPI data was then exported, and visualized using Cytoscape.

### 2.4 Age-related cox regression model construction

Currently, the least absolute shrinkage and selection operator (LASSO) regression is the most employed machine-learning algorithm for the establishment of a diagnostic model. A regularization method was used to solve overfitting during curve fitting to enhance the accuracy of the model. To enhance the accuracy of the model, we employed the glmnet R package (Simon et al., 2011) to establish a model based on age-associated genes (family = “binomial”).

Patients with PTC in the TCGA database were separated into a training set of 244 cases and a validation set of 244 cases at a 1:1 ratio. To assess the predictive power of gene expression on progression-free survival (PFS), we conducted univariate Cox regression, LASSO Cox regression, and multivariate Cox regression analyses to further screen for prognosis-associated genes, through which a prognostic model was established based on TCGA datasets. First, we used univariable Cox proportional regression to test the associations between the expression of each differential gene and PFS, and only retained the genes with a *p* value ≤ 0.05. We then removed multicollinearity using the LASSO algorithm and determined meaningful variables in the univariate Cox regression analysis. Moreover, to obtain more accurate self-contained predictors for outcomes, we used multivariate Cox regression analysis along with stepwise regression for the final selection. Eventually, the formula for calculating risk scores was established by considering the expression of optimized genes and correlation-estimated Cox regression coefficients. Risk score = (expression gene1 × coeff gene1) + (expression gene2 × coeff gene2) + … (expression gene × coeff gene). The samples were separated into high- and low-risk groups based on their risk scores. We also analyzed the PFS of the validation set using the Kaplan–Meier method and log-rank test. A time-dependent receiver operating characteristic (ROC) curve was employed to assess the predictive capacities, followed by the calculation of the area under the curve (AUC) to analyze the accuracy of the prediction model.

### 2.5 Functional enrichment analysis

Gene ontology (GO) analysis, a popular and valuable approach for wide-ranging functional enrichment research, comprises cellular components (CCs), molecular functions (MFs), and biological processes (BPs). Kyoto Encyclopedia of Genes and Genomes (KEGG) is a comprehensive database for analyzing the relationships between genomes, diseases, biological pathways, drugs, and chemical substances. In this study, we used the R package clusterProfiler (Yu et al., 2012) to conduct GO and KEGG analyses of DEGs.

Moreover, gene set enrichment analysis (GSEA) (Subramanian et al., 2005) was used to investigate the differential biological processes according to the TCGA-PTC dataset gene expression profile data from the high- and low-risk groups. GSEA is an extensively used bioinformatics analysis tool for detecting discrepancies in statistical significance and concordance between two biological states and is also widely utilized for estimating alterations of the path and biological process activity in expression profile samples. We downloaded gene sets from the Molecular Signatures Database (MSigDB) (Liberzon et al., 2015) c2.cp.v7.2. symbols.gmt for GSEA analysis. *p* value < 0.05 was considered statistically significant.

### 2.6 Survival analysis and clinical correlations analysis

Based on the multivariate analysis results, we integrated multiple predictors and assigned them in a certain proportion, and then graphically visualized the predictive inter-relationships among all the variables on disease outcome. Based on the risk score, sex, and pathologic stage, we implemented multivariate Cox regression to predict the incidence of PTC progression, constructed a nomogram, and further evaluated the model’s predictive capacity.

Subsequently, to study the association between gene expression and PFS, PTC patients were categorized into high- and low-expression groups based on the median expression values for each gene, followed by Kaplan-Meier survival analysis and log-rank tests. Furthermore, we examined the expression levels of key genes in patients with PTC at different pathological stages to further identify their associations (Supplementary Table S2).

### 2.7 Correlation analysis of immune infiltration

We retrieved immune-related gene sets (Supplementary Material S1) from the ImmPort Database (https://www.immport.org) and used the “GSVA” package (Hänzelmann et al., 2013) to apply a single-sample GSEA (ssGSEA) analysis. Next, we estimated the scores of the 16 immunology-associated gene sets and compared their differences between the high- and low-risk groups and among the key genes.

CIBERSORT (Newman et al., 2015) is a bioinformatics method used to deconvolve the transcriptome expression matrix according to the guidelines for linear support vector regression and estimation of the composition and abundance of immune cells within a mixed cell population. Ultimately, we obtained the immune cell infiltration matrix by uploading the TPM-normalized gene expression matrix to CIBERSORT, combining the gene signature matrix (LM22), and filtering the output to include only significant samples with a *p* value < 0.05. Later, bar graphs were constructed in R utilizing the “ggplot2” package to exhibit the distribution of various kinds of infiltrating immune cells in each sample and the expression of different immune cells in the two differential risk groups.

### 2.8 Drug sensitivity analysis

The mRNA expression profiles of NCI-60 cell lines and drug activity data were obtained from CellMiner (http://discover.nci.nih.gov/cellminer). CellMiner is an online tool for researchers to take advantage of transcript and drug response data in NCI-60 cell line sets, which contain abundant resources for genomes and pharmacology, and was edited by the U.S. National Cancer Institute. Specifically, it was enriched in the transcriptomic expression levels of 22,379 genes, 360 microRNAs, and drug responses of 20,503 compounds. We calculated the associations between the expression levels of key genes and drug sensitivity using Pearson correlation analysis. Differences were considered statistically significant at *p* < 0.05.

### 2.9 Statistical analyses

All statistical analyses were performed using R software (https://www.r-project.org/, version 4.0.2). For continuous variables, the Mann-Whitney U test (Wilcoxon rank-sum test) was applied for comparisons between two groups, and the Kruskal–Wallis test was applied for comparisons of more than two groups. We constructed ROC curves and calculated the AUC for an improved assessment of the model using the pROC package in R (Sing et al., 2005). Each *p*-value was two-sided, and the significance level was set at *p* < 0.05.

## 3 Results

### 3.1 Technology roadmap

**FIGURE 1:**
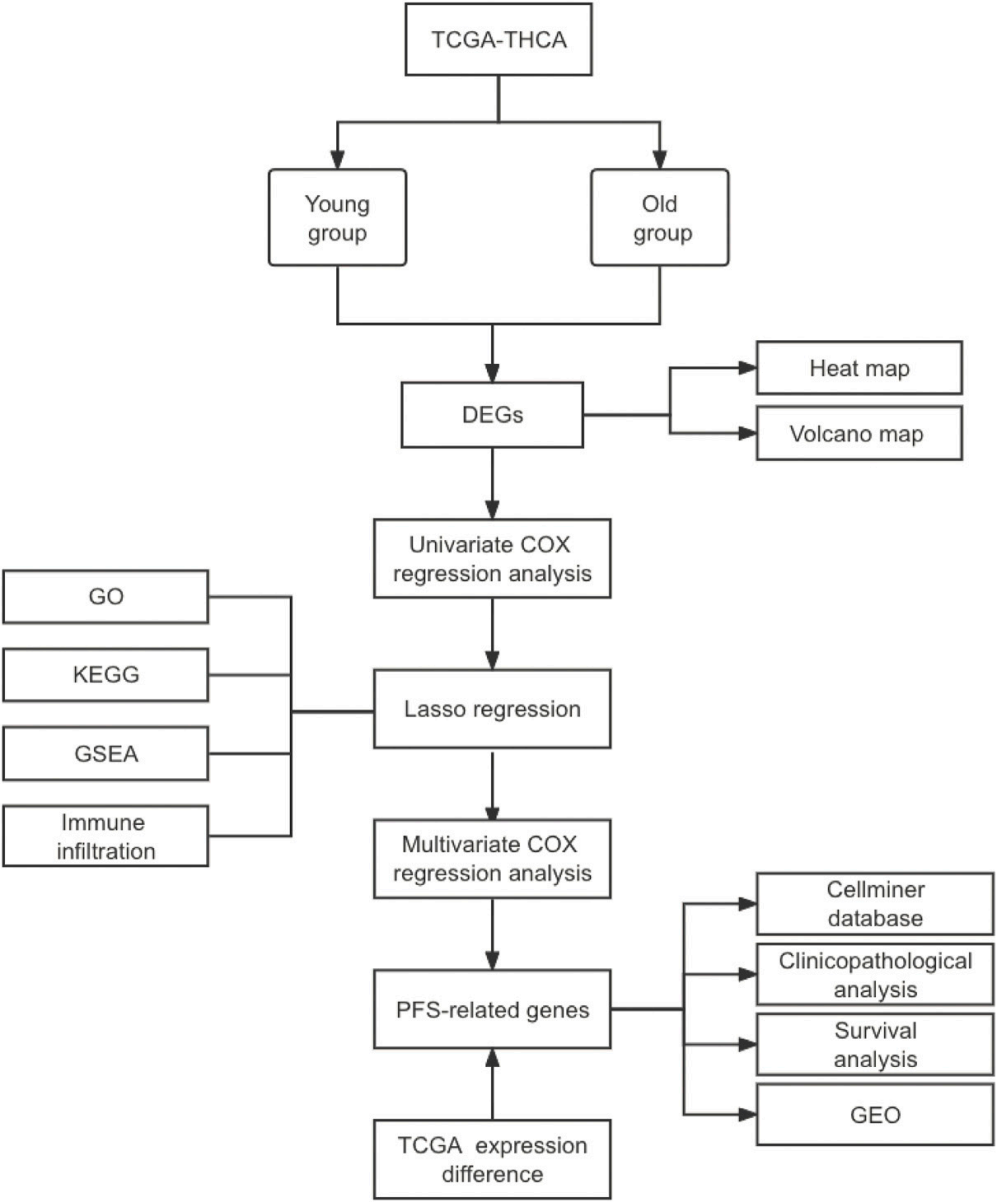
Technology roadmap.

The Technology roadmap of this study is shown in Figure 1.

### 3.2 Identification of the age-associated genes in papillary thyroid carcinoma

**FIGURE 2:**
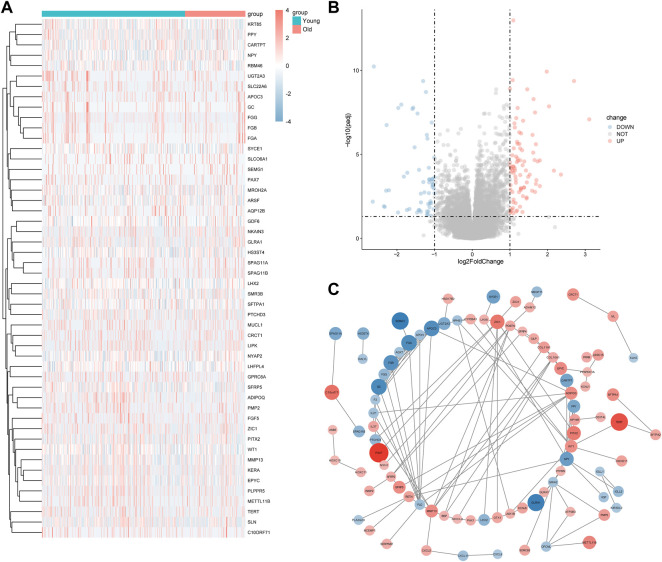
Age-related genes in papillary thyroid carcinoma (PTC). **(A)** In the heatmap, the ordinate is the number of differentially expressed genes (DEGs), and the abscissa shows the ID of the patients; red indicates high expression and blue indicates low expression; the pink bar represents tumor sample, whereas the blue bar represents healthy tissue. **(B)** The abscissa is log2 (FC value) and the ordinate is–log10 (Adjusted *p* value). The red node indicates upregulated DEGs, the blue node indicates downregulated DEGs, and the gray node indicates non-DEG. **(C)** The red dot represents highly expressed genes in the elderly group, while the blue dot represents highly expressed genes in the young group, and the size of the dot represents the absolute value of log2FC in the protein-protein interaction graph.

To analyze the impact of gene expression values in PTC tissues relative to healthy tissues, we identified 146 DEGs (88 upregulated and 58 downregulated genes) in the elderly group relative to the younger group using the DESeq2 package for differential expression analysis. The heat map was plotted using the top 25 genes with the smallest and the largest log2FC values in Figure 2A, and the differential gene expression is shown in a volcano plot in Figure 2B. The STRING database was used to draw a PPI network graph for 146 differentially expressed age-related genes (Figure 2C).

### 3.3 Age-related cox regression model construction

**FIGURE 3:**
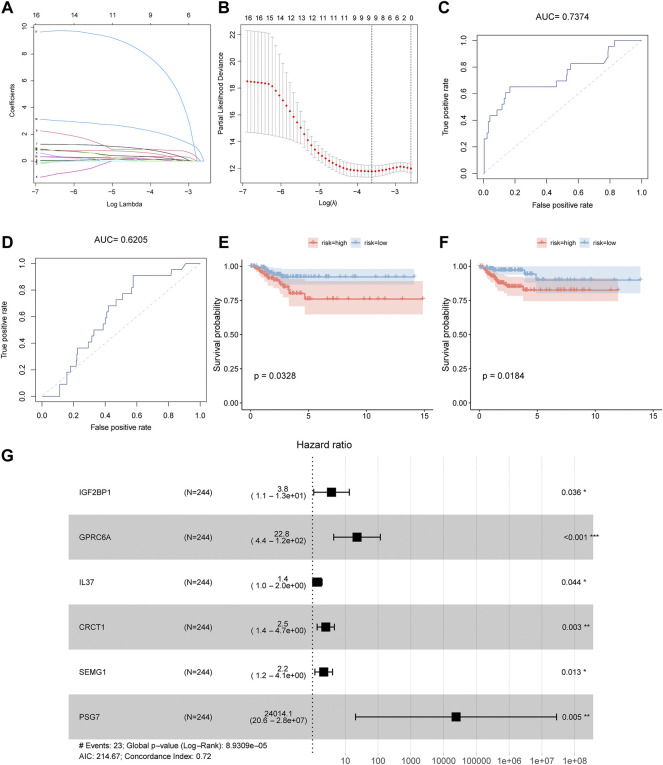
The LASSO regression model. **(A)**, **(B)** The LASSO-Cox regression model construction based upon the differential expressions of age-associated genes. **(C)**, **(D)** ROC curves for predicting survival according to the risk scores in the training and validation cohorts, respectively. **(E)**, **(F)** Survival curves of the high- and low-risk groups in the training and validation cohorts, respectively. **(G)** Multivariate Cox regression analysis forest map.

First, we assigned 488 patients with PTC from TCGA to 244 cases in the training set and 244 cases in the validation set. Next, we obtained a total of 17 variables by performing a univariate Cox regression analysis to examine the relationship between the variables and PFS in the training set. Then, we performed LASSO regression analysis (Figures 3A,B) according to the nine genes obtained by univariate Cox regression, further removed variables with higher multicollinearity, and identified nine key genes associated with PFS (FCRLB, TERT, IGF2BP1, PLPPR5, GPRC6A, IL37, CRCT1, SEMG1, and PSG7). Finally, we obtained six independent factors associated with PFS (IGF2BP1, GPRC6A, IL37, CRCT1, SEMG1, and PSG7) according to the multivariate regression analysis of the nine genes and further validated them using the validation set. The risk scores of patients with PTC were calculated using the following formula: Risk score = IGF2BP1 * 1.34 + GPRC6A * 3.13 + IL37 * 0.343 + CRCT1 * 0.932 + SEMG1 * 0.785 + PSG7 * 10.086. We separately conducted ROC curve analysis of the risk score to predict PFS in the training and validation sets, with the results including an AUC of 0.7374,0.6205 and *p* value for survival curves of 0.0328,0.0184, respectively (Figures 3C–F). Collectively, the LASSO regression model based on the six key genes performed excellently in evaluating the PFS of patients with PTC, and the forest plot intuitively illustrated the associations between the six key genes and hazard ratios (Figure 3G).

### 3.4 Functional enrichment analysis

**FIGURE 4:**
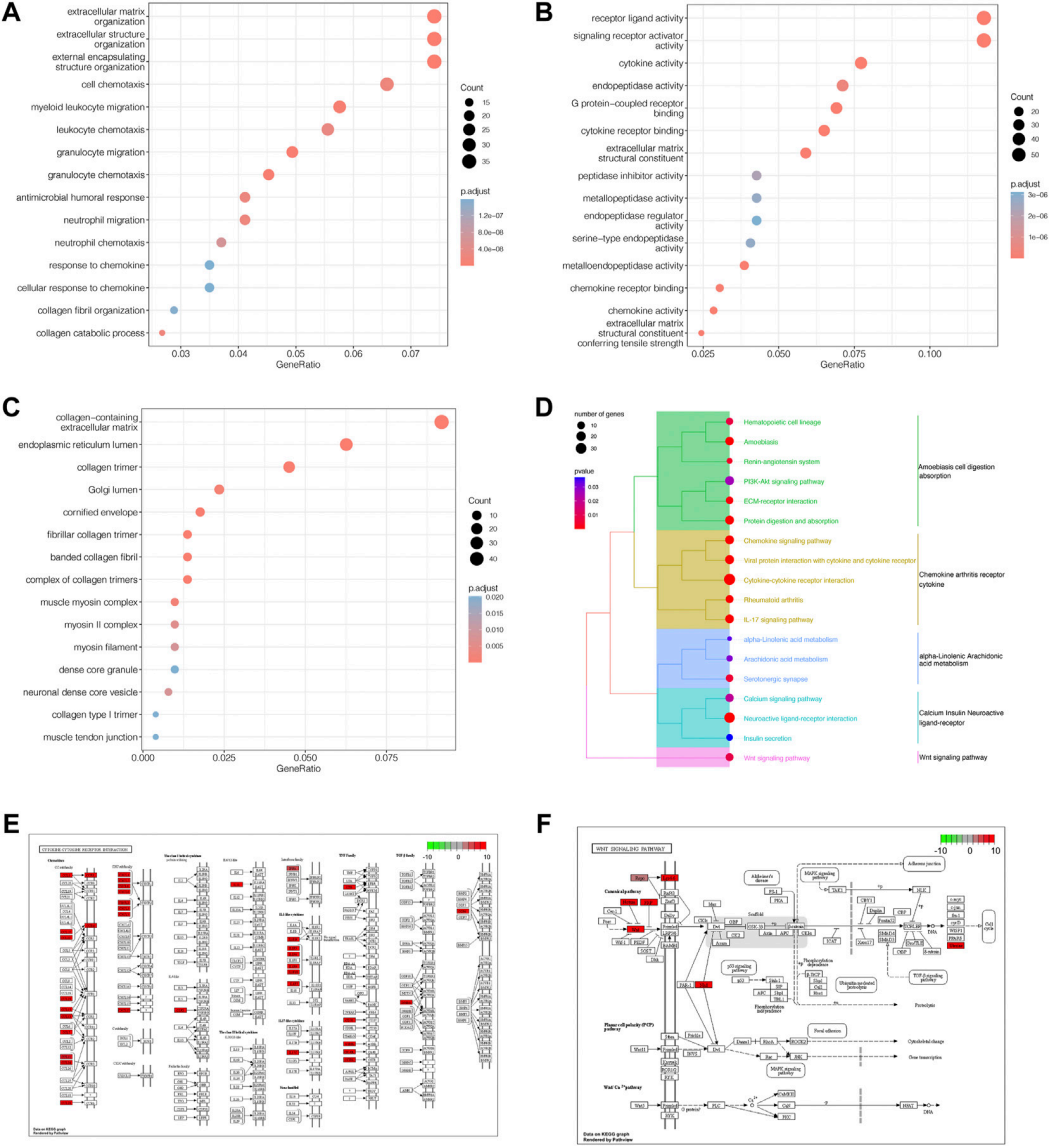
GO and KEGG pathway enrichment analysis. **(A)** Bubble diagram of GO enrichment analysis for biological processes. **(B)** Bubble diagram of GO enrichment analysis for molecular functions. **(C)** Bubble diagram of GO enrichment analysis for cellular components. In all the graphs above, the ordinate represents the number of genes, and the abscissa represents the GO term. The node size indicates the number of enriched genes whereas node color denotes the Padj value. **(D)** Five clusters were observed in the clustering dendrogram of the KEGG pathway enrichment analysis. The node size indicates the number of genes and node color indicates the *p* value. The ordinate indicates the KEGG term. **(E)**, **(F)** Cytokine-cytokine receptor interaction signaling pathway map and WNT-signaling pathway map.

We calculated the risk scores for all patients with PTC from TCGA based on the coefficients obtained from the multivariate Cox regression analysis. As hypothesized, they were categorized into high- and low-risk groups based on the median risk score. To characterize DEGs, we analyzed three kinds of GO functional annotations, including BP, MF, and CC categories, followed by the related KEGG pathway (Supplementary Tables S3–S5). For BPs, DEGs were mostly concentrated in the extracellular matrix organization, extracellular structure organization, external encapsulating structure organization, and granulocyte migration. Regarding molecular function, DEGs were primarily localized to receptor-ligand activity, signaling receptor activator activity, cytokine activity, and extracellular matrix structural constituents. As for CCs, DEGs clustered in the collagen trimer, collagen-containing extracellular matrix, endoplasmic reticulum lumen, and fibrillar collagen trimer. KEGG enrichment analysis indicated that these DEGs were centralized in cytokine-cytokine receptor interaction, viral protein interaction with cytokine and cytokine receptor, the interleukin 17 (IL-17) signaling pathway, and the Wingless and Int-1 (WNT) signaling pathway (Figures 4A–D). Meanwhile, we displayed the cytokine-cytokine receptor interaction pathway and WNT signaling pathway as representatives of the remarkably enriched pathways (Figures 4E,F, Supplementary Table S6).

### 3.5 Gene set enrichment analysis

**FIGURE 5:**
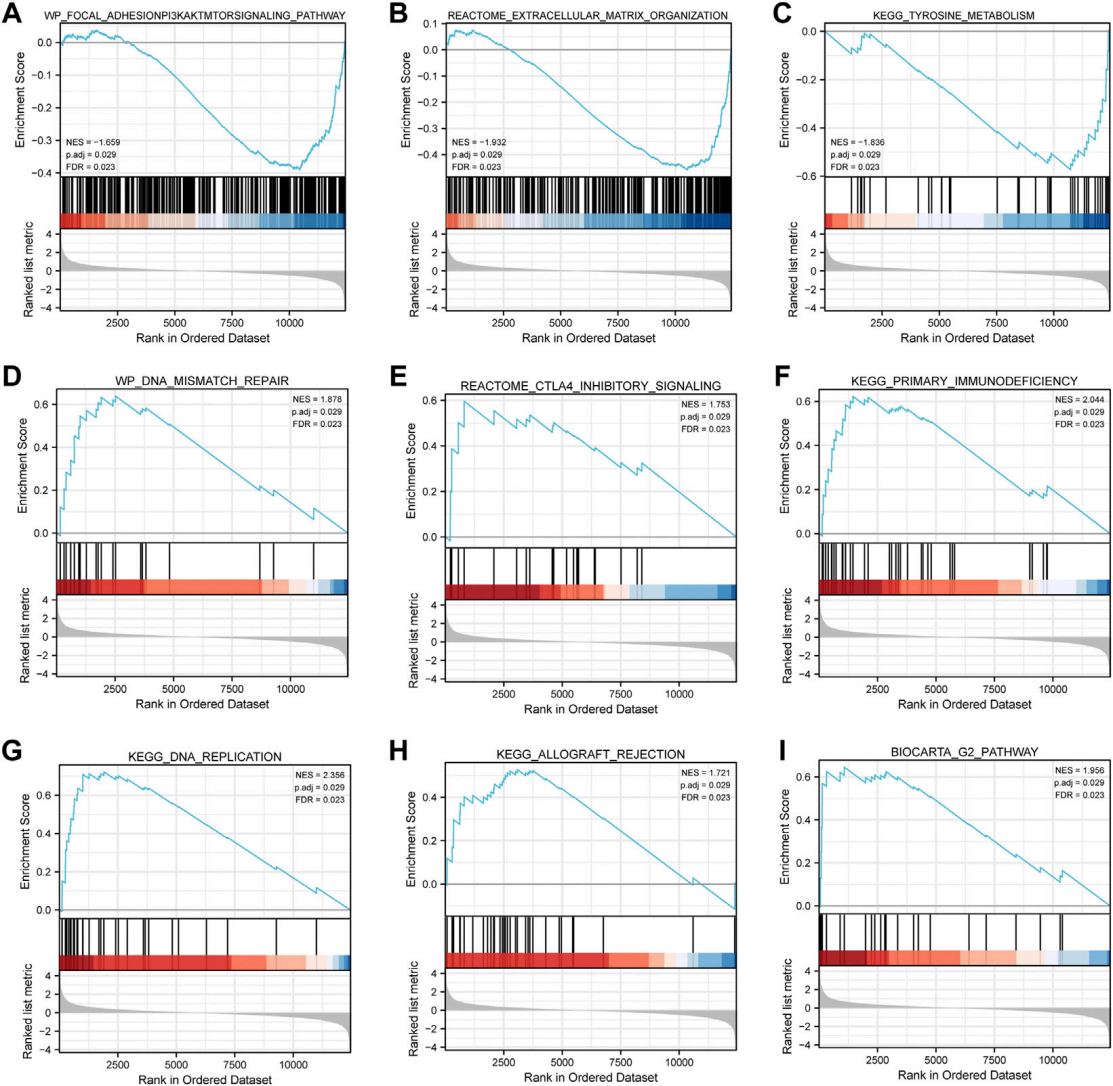
Gene set enrichment analysis. **(A–C)** Biological functions, such as the focal adhesion-PI3K-AKT-mTOR signaling pathway, extracellular matrix organization, and tyrosine metabolism, were markedly concentrated in the low-risk group. (**D–I**) Other biological functions, such as DNA mismatch repair, CTLA4 inhibitory signaling, primary immunodeficiency, DNA replication, allograft rejection, and the G2 pathway, were markedly concentrated in the high-risk group.

For the in-depth identification of the biological pathways associated with PFS, we used GSEA to analyze the differences between the high- and low-risk groups. Biological functions, such as the focal adhesion-PI3K-AKT-mTOR signaling pathway (Figure 5A), extracellular matrix organization (Figure 5B), and tyrosine metabolism (Figure 5C), were remarkably enriched in the low-risk group, while other biological functions, such as DNA mismatch repair (Figure 5D), CTLA4 inhibitory signaling (Figure 5E), primary immunodeficiency (Figure 5F), DNA replication (Figure 5G), allograft rejection (Figure 5H), and the G2 pathway (Figure 5I), were strikingly concentrated in the high-risk group (Supplementary Material S2).

### 3.6 Correlation analysis of immune infiltration

**FIGURE 6:**
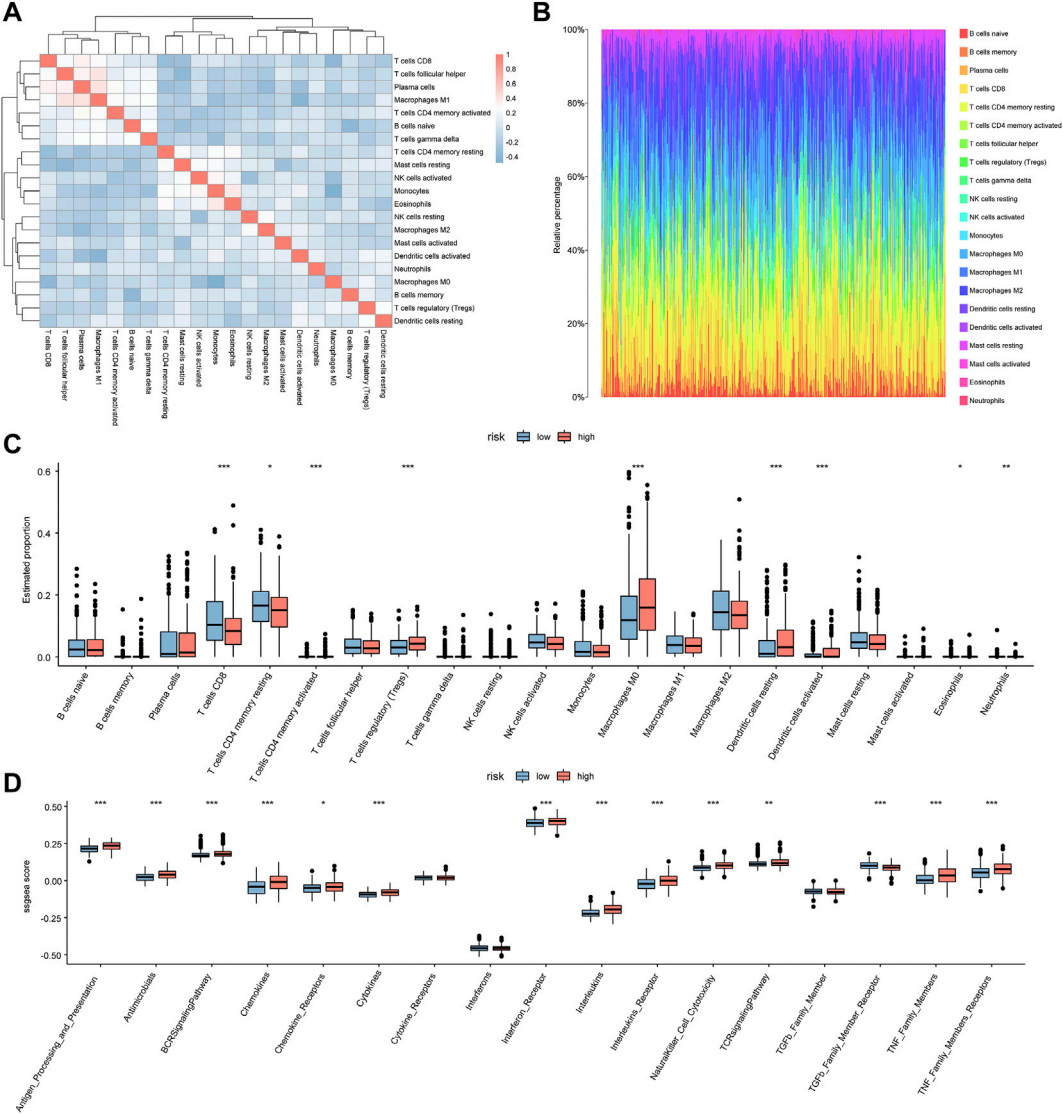
Correlation analysis of immune infiltration. **(A)** In the heatmap for correlation among immune cells, red indicates positive correlation whereas blue indicates negative correlation. **(B)** In the graph for the distribution of immune cell infiltration of patients, the abscissa represents different samples from TCGA and the ordinate represents the proportion of immune cells. **(C)** The box plot indicates the difference in the infiltration abundance of immune cells between the two risk groups; the horizontal axis represents the immune cells whereas the vertical axis represents the infiltration abundance of different immune cells; red indicates the high-risk group while blue indicates the low-risk group. **(D)** In the box plot of the differences in the scores of immune gene sets between the two risk groups, the horizontal axis shows the different immune-related gene sets whereas the vertical axis indicates the ssGSEA score, and red indicates the high-risk group while blue indicates the low-risk group.

First, the CIBERSORT algorithm was used to calculate the degree of immune cell infiltration for PTC patients. Next, we demonstrated the distribution of immune cell infiltration in every patient (Figure 6B) and the correlation among different immune cells (Figure 6A). We further displayed differential immune cell infiltration between the high- and low-risk groups in box plots. Accordingly, we concluded that nine of the 21 types of immune cells had a significant relationship with differential immune cell infiltration, and CD8^+^ T cells decreased significantly, while regulatory T cells (Tregs) were significantly elevated in the high-risk group (Figure 6C), suggesting that age-related key genes could be significantly associated with these immune cells. In addition, 14 immune-associated gene sets showed remarkable discrepancies (Figure 6D).

### 3.7 Prognostic model of age-related genes

**FIGURE 7:**
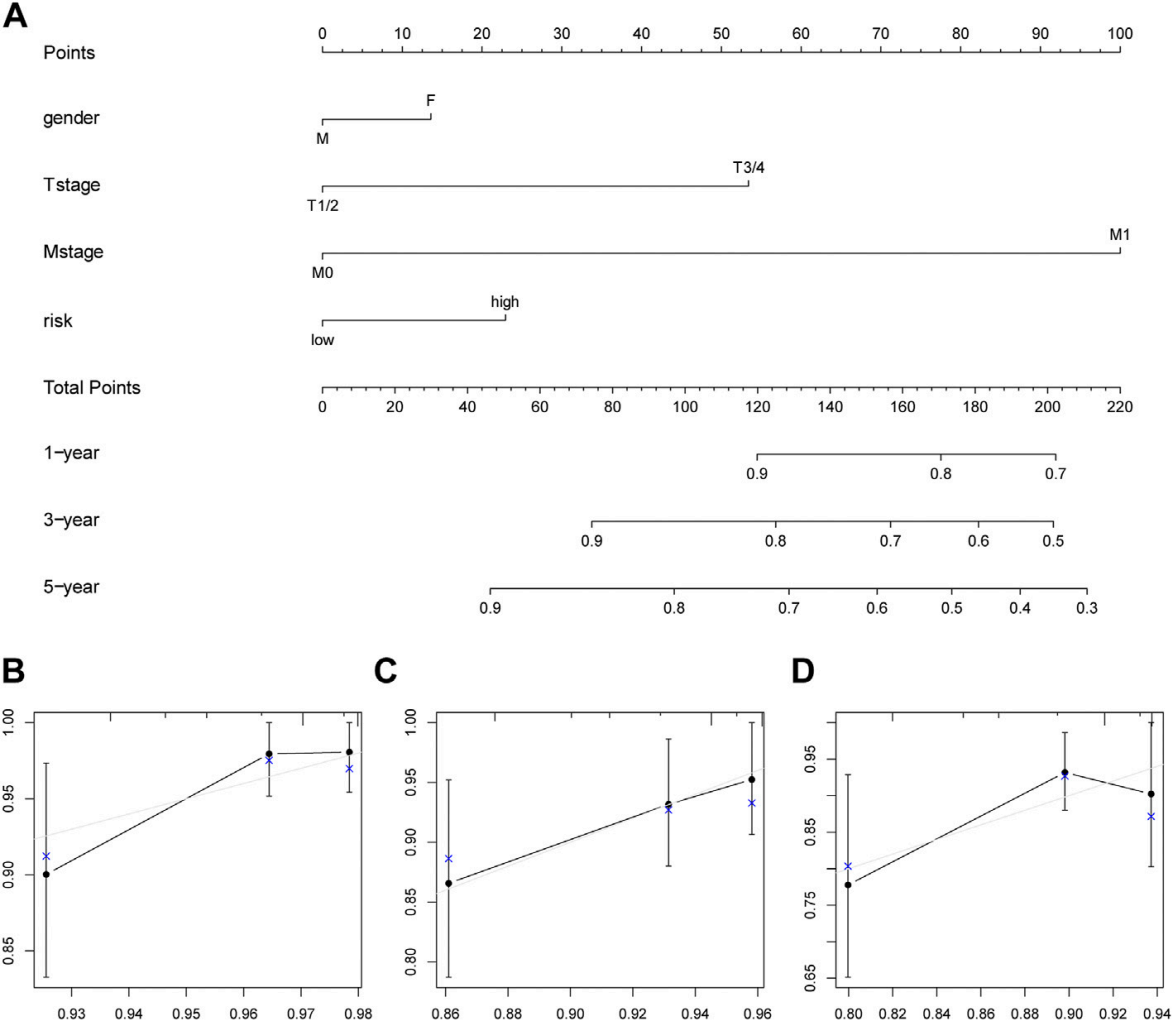
The predictive model. **(A)** Nomogram constructed based on risk score, T stage, M stage, and gender of the PTC patients. **(B–D)** Calibration plots of the nomogram for predicting the one-, three- and five-year survival, respectively.

According to the risk score, T staging, M staging, and sex, we constructed a model to predict PFS in patients with PTC, plotted a nomogram (Figure 7A), and estimated the model. In addition, we constructed calibration plots to predict the one-, three-, and five-year PFS (Figures 7 B–D), suggesting that this model was capable of accurately predicting PFS in PTC patients to some extent, with better predictive competence for tumor progression at three years.

### 3.8 Expression differences of age-related genes in the cancer genome atlas

**FIGURE 8:**
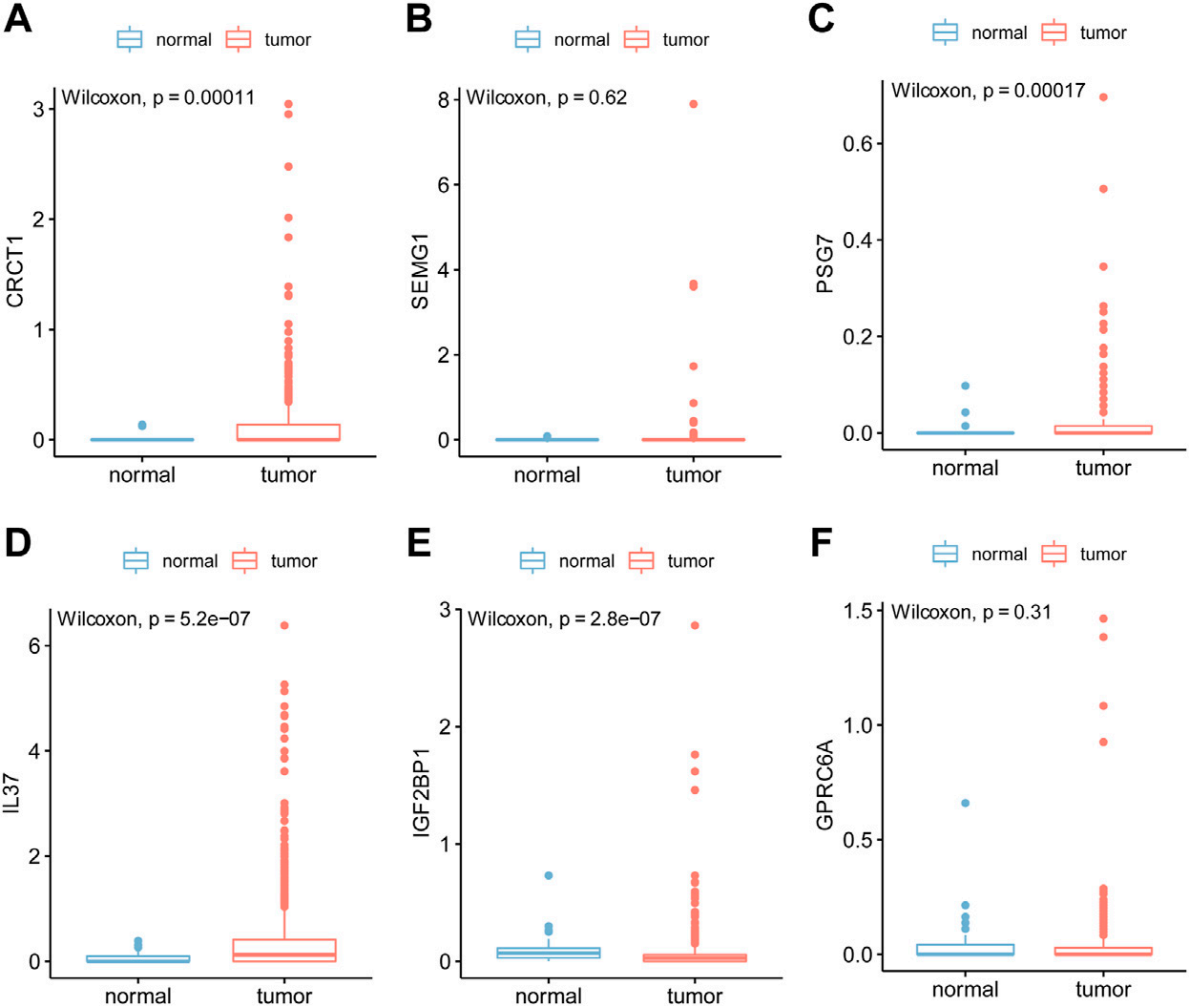
The box plot of differential gene expression in TCGA. Box plots for differential expressions of key genes, including IGF2BP1, GPRC6A, IL37, CRCT1, SEMG1, and PSG7, between the PTC group and healthy group in the TCGA database. The blue represents normal samples from healthy people and red represents tumor samples from patients with PTC. *P* value <0.05 was considered statistically significant (Figures 8A–F).

Of the six genes (IGF2BP1, GPRC6A, IL37, CRCT1, SEMG1, and PSG7) for which we analyzed the differential expression between tumor tissues and healthy tissues in TCGA (Figure 8), four genes (CRCT1, PSG7, IL37, and IGF2BP1) showed notable differences (Figures 8A,C–E).

### 3.9 Drug sensitivity analysis

**FIGURE 9:**
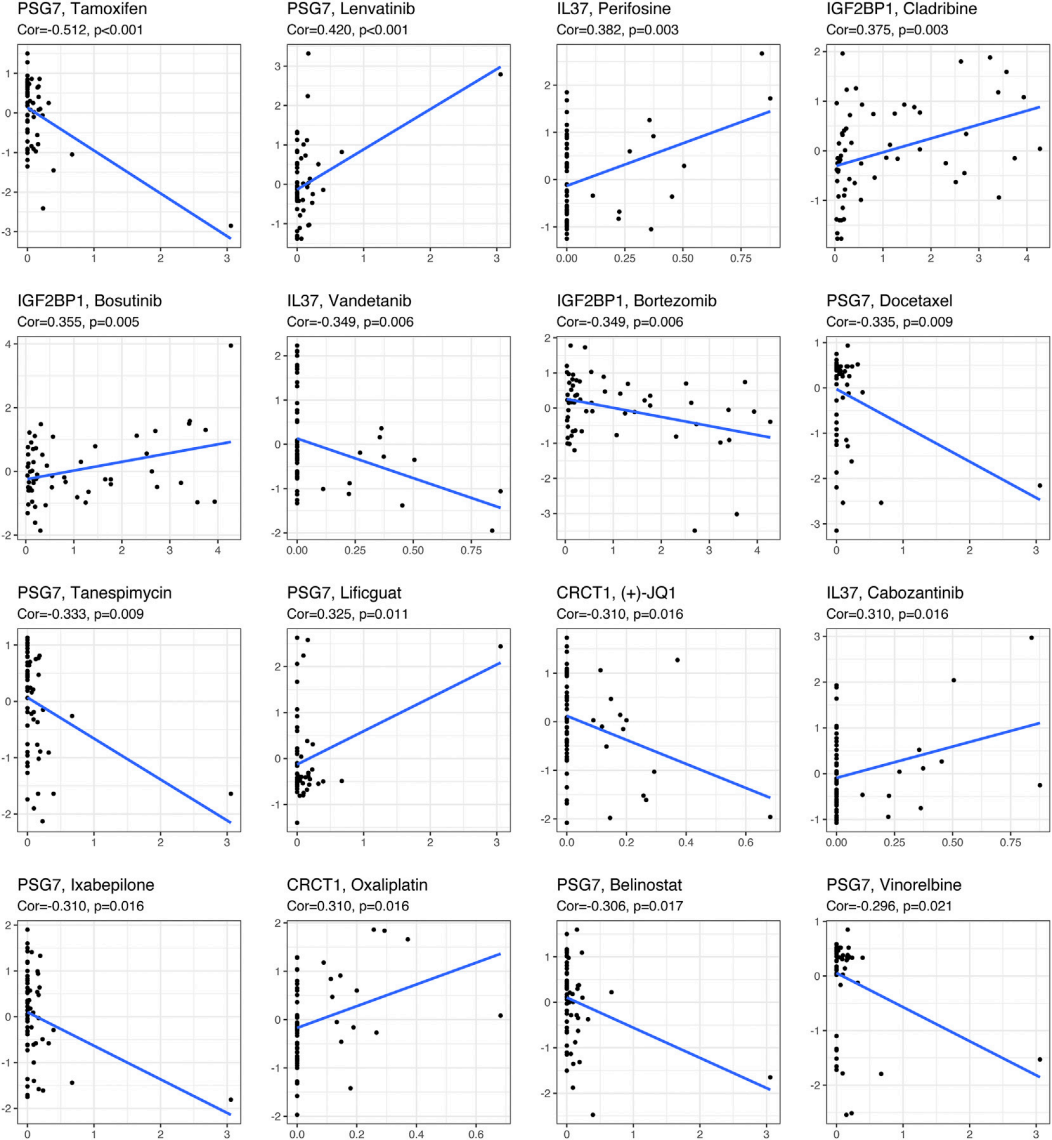
Drug sensitivity analysis of key genes (CRCT1, PSG7, IL37, and IGF2BP1) using the CellMiner database.

The CellMiner database was used to investigate the relationship between the expression levels of four genes (CRCT1, PSG7, IL37, and IGF2BP1) and drug sensitivity. Immediately after, we identified the 16 drugs with the lowest *p* value in the correlation analysis (Figure 9). With respect to the associations of drug sensitivity with the four age-related prognostic genes, PSG7 had a positive correlation with lenvatinib, as well as Lificiguat, but a negative correlation with docetaxel, tamoxifen, tanespimycin, belinostat, vinorelbine, and ixabepilone. There was a positive association between IGF2BP1 and cladribine and bosutinib, but a negative association between IGF2BP1 and bortezomib. IL-37 exhibited a positive relationship with perifosine and cabozantinib, but a negative relationship with vandetanib. CRCT1 was positively correlated with oxaliplatin but negatively correlated with (+)-JQ1.

### 3.10 Correlation analysis and validation between gene expression and clinicopathologic characteristics

**FIGURE 10:**
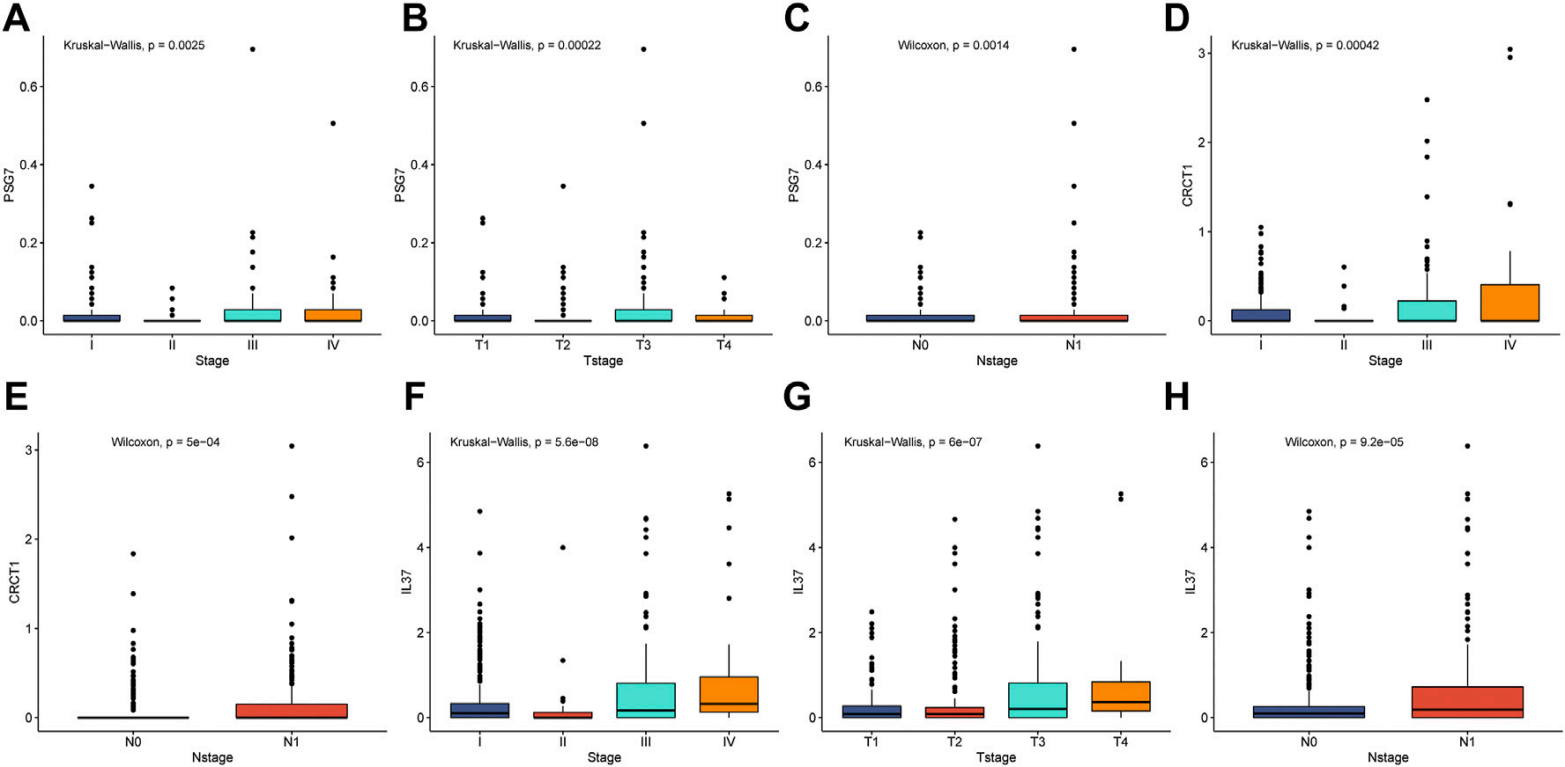
The correlation analysis of clinicopathological factors. **(A–C)** PSG7 expression values had a strong correlation with pathological stage, T stage, and N stage for PTC patients. **(D–E)** CRCT1 expression levels showed a remarkable association with pathological stage and N stage for PTC patients. **(F–H)** IL-37 expression values had a significant relationship with pathological stage, T stage, and N stage for PTC patients.

Using the data of PTC patients in the TCGA database, we evaluated the associations of the key genes and four clinical features (pathological stage, T stage, N stage, and M stage) (Figures 10A–H). PSG7 was remarkably correlated with clinicopathological parameters (pathological stage, T stage, and N stage), and high PSG7 expression was associated with the progression of the clinical stage (Figures 10A–C). CRCT1 expression was significantly related to the pathological stage and N stage of patients (Figures 10D,E). IL37 expression was strongly correlated with pathological stage, T stage, and N stage, implying that its high expression was closely correlated with a higher clinical stage (Figures 10F–H).

**FIGURE 11:**
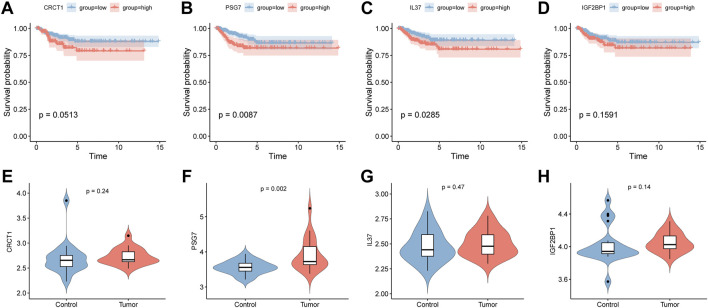
Survival analysis for key genes in TCGA and expression comparisons for key genes using the DEO database. **(A–D)** The Kaplan–Meier analyses of progression-free survival between the high- and low-expression groups for the key genes (CRCT1, PSG7, IL-37, and IGF2BP1). **(E–H)** The expression values of the key genes (CRCT1, PSG7, IL-37, and IGF2BP1) in the PTC and healthy group in the GEO database.

According to the median expression of CRCT1, PSG7, IL37, and IGF2BP1, we dichotomized the samples into high- and low-expression groups using the TCGA dataset. We further carried out Kaplan–Meier analysis and log-rank tests to evaluate the association between gene expression and PFS in PTC patients (Figures 11A–D). Overall, patients with a high expression of key genes had a shorter PFS. We representatively showed that PSG7 and IL37 were statistically significant in the different groups (*p* = 0.0087 and *p* = 0.0285, respectively). Meanwhile, we compared the gene expression of CRCT1, PSG7, IL37, and IGF2BP1 between the PTC and healthy groups from GEO data (Figures 11E–H) and found that PSG7 was statistically significant in the two groups at *p* = 0.002.

### 3.11 Correlation analysis between the key genes and immune infiltration

**FIGURE 12:**
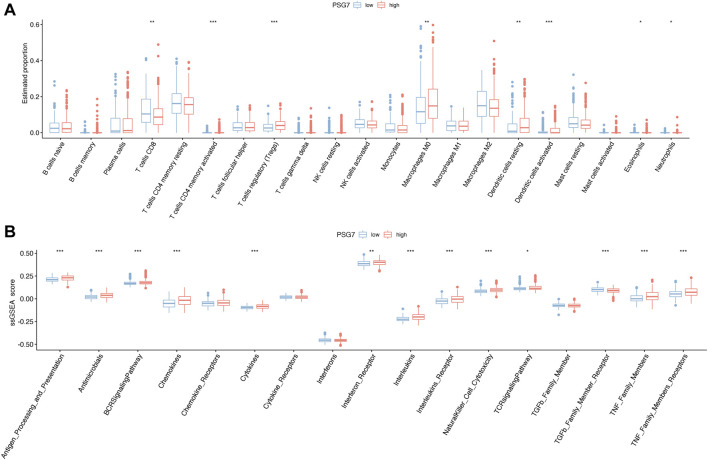
Correlation analysis of immune infiltration. **(A)** The box plot depicted the abundance differences in immune cell infiltration between high- and low-expression PSG7 groups. Horizontal axes represent immune cells and vertical axes represent the infiltration abundance of different immune cells. **(B)** Box plot of the different scores of immune gene sets between high- and low-expression PSG7 groups. The horizontal axis indicates different immune-related gene sets, and the vertical axis indicates ssGSEA scores. In both figures, red denotes the high-expression group and blue denotes the low-expression group.

To further investigate the relationship between the PSG7 expression level and tumorous immunity, we initially separated the PTC samples into high- and low-expression groups using the median value of PSG7 expression. Subsequently, we displayed the differentially infiltrated immune cells between the two different expression groups in box plots. Interestingly, we discovered that eight of the 21 types of immune cells were related to the differentially infiltrated immune cells, and CD8^+^ T cells decreased significantly, while Tregs increased significantly in the high-expression group (Figure 12A), suggesting that PSG7 may exert a nontrivial effect on PTC deterioration. Additionally, 13 gene sets were remarkably different from the immune-related gene sets (Figure 12B).

## 4 Discussion

Despite a generally favorable prognosis, emerging evidence suggests that prognosis worsen with increasing age in patients with PTC (Gosain et al., 2018; Ito et al., 2019b), and the underlying mechanism remains unclear. The cut-off value of age at diagnosis used for staging increased from 45 to 55 in the latest AJCC 8th staging system for differentiated thyroid carcinoma, which can allow low-risk patients to avoid aggressive surgical resections and postoperative therapies, improve disease management strategies, and reduce psychosocial and financial burdens (Pontius et al., 2017; Shteinshnaider et al., 2018). However, age at diagnosis alone does not appear to be an ideal guideline for survival prognosis judgment, precise risk stratification, and scientific therapy guidance, without considering specific molecular markers (Verburg et al., 2018). Hence, a novel strategy to explore the exact molecular mechanism of PTC progression with age will provide appreciable benefits for individualized treatment. Through bioinformatics analysis, we concluded that some meaningful biomarkers, altered pathways, and reshaped tumor immune microenvironments are involved in PTC progression. We demonstrated for the first time that PSG7 may be inextricably linked with tumorigenesis and may be a promising predictor for prognosis estimation in PTC.

First, we screened 88 upregulated and 58 downregulated DEGs from the young and elderly groups of PTC samples from TCGA with a cutoff age of 55 years, which is consistent with the latest AJCC 8th staging system for thyroid carcinoma (Perrier et al., 2018). We obtained six independent prognostic predictors (IGF2BP1, GPRC6A, IL-37, CRCT1, SEMG1, and PSG7) associated with PFS in PTC patients, based on which GO, KEGG, GSEA, immune infiltration, and age-related prognostic model analyses revealed that each key gene was intimately linked to PTC development. Interestingly, PSG7, CRCT1, and IL-37 were statistically significant in the clinical correlation analysis. Furthermore, only samples in the PSG7 high-expression group and IL-37 high-expression group had statistically shorter PFS in the survival analysis, and PSG7 was statistically significant in the differential expression analysis between PTC and healthy groups in the GEO database. In addition, the reshaped tumor immune microenvironment participated in PTC development in the PSG7 high-expression group. Taken together, PSG7 may have important implications in the pathogenesis of PTC and may serve as the most promising age-related prognostic marker for PTC in our study.

Pregnancy-specific glycoprotein (PSG) genes belong to the carcinoembryonic antigen (CEA) gene family of the immunoglobulin gene superfamily. It is known that there are at least ten members (PSG1–PSG9, PSG11) encoding intimately associated secreted glycoproteins in humans, which have the highest contents of all proteins secreted by the fetal trophoblast to maintain a viable pregnancy (Moore and Dveksler, 2014; Zimmermann and Kammerer, 2021). Based on sequence homology, the CEA gene family can be separated into two subgroups: PSG and CEA (Streydio et al., 1988). CEA has been extensively studied and used as a cancer marker (Hester et al., 2021; Moretto et al., 2021; Taheri et al., 2022), whereas PSG, especially PSG7, has been relatively poorly studied in the field of oncology, (Su et al., 2020). Unlike other PSGs, PSG7 is characterized by an unblocked N-terminus, which may indicate special biological functions worthy of further research (Khan and Hammarström, 1990). As the most promising key gene in our study, PSG7 had a positive association with drug susceptibility to lenvatinib and lificiguat, which is highly consistent with the actual clinical situation. Lenvatinib, a US Food and Drug Administration approved oral drug, is widely used to treat adult well-differentiated thyroid cancer. In addition to its definite efficacy in treating adult patients with drug resistance, recurrence, metastasis, and rare pathological subtype (Tori and Shimo, 2018; Ito et al., 2019a; Takinami and Yokota, 2020), lenvatinib shows potential for application in the treatment of children with PTC who are intractable or not tolerant to conventional therapy (Mahajan et al., 2018). Furthermore, it brings new hope to patients with advanced unresectable PTC, for whom this novel option may increase the opportunity for surgical resection (Iwasaki et al., 2020). In addition, lificiguat is the first generation of soluble guanylyl cyclase stimulators, which is currently under investigation in the field of cardiovascular diseases and beyond (Sandner et al., 2021). In short, drug sensitivity analysis of key genes will bring substantial benefits to individualizing therapeutic recommendations for specific patients with PTC.

As mentioned earlier, PSG7 is the only key gene highly expressed in both PTC tumor tissues from TCGA and GEO compared with healthy tissues. More intriguingly, PSG7 had dramatically close correlations with pathological stage, T stage, and N stage for PTC patients, and high expression of PSG7 consistently suggested a worse prognosis. The maternal serum concentration of PSG gradually increases as pregnancy progresses under normal circumstances (Lin et al., 1976), and the relationship between PSG and tumorigenesis has attracted the attention of researchers in recent years. As the fifth member of the PSG family detected in the fetal liver (Khan and Hammarström, 1990), PSG7 was expected to interact with the prognostic lncRNAs (CTD-2218G20.2) in gastric cancer, but the actual mechanism remains elusive (Su et al., 2020). Although less research has been done on PSG7 thus far, several significant advances have been made regarding the other members of its family. Among all the PSGs, higher expression of PSG9 was confirmed to have significantly stronger associations with poor clinical and pathological features, as well as worse survival prognoses in several tumors. In hepatocellular carcinoma, two studies consistently demonstrated that significantly increased PSG9 protein in the plasma exerted an important regulatory impact on tumor proliferation and progression and served as a self-contained biomarker for predicting prognosis (Rong et al., 2017; Rong et al., 2019). Similarly, the elevated expression of PSG9 in plasma specimens and tumorous tissue was strongly associated with poor clinical and prognostic parameters, such as metastatic lymph node tissues, distant metastasis, and shorter DFS in breast cancer patients (Liu et al., 2020). In addition, the mRNA level of PSG9 significantly increased with an increase in the transcription factor AP-2α (TFAP2A) in lung adenocarcinoma, which predicts poorer OS and PFS, probably because of the promotion of tumor metastasis (Xiong et al., 2021). In cervical cancer, PSG1 was detected in 90% of precancerous lesions and all cancerous serum specimens, whereas it was undetectable in healthy women, which may provide clues for its carcinogenesis, immunological memory, and immunotolerance (Rodríguez-Esquivel et al., 2020). In addition, PSG2 and PSG5 levels were elevated to different degrees in cervical cancer specimens in comparison to identical healthy tissues, the promoter regions of which were bound by Krüppel-like factors to regulate cellular proliferation and differentiation (Marrero-Rodríguez et al., 2018). For stomach adenocarcinoma, PSG6 is one of the seven prognostic genes associated with increased mortality, either individually or in combination (Wang et al., 2020). Owing to its genomic and functional complexity, the study of aberrant expression of PSG contributing to cancer is weak, particularly in PTC, and the functions of PSG7 have never been studied so far, but future studies using more rigorous designs and well-validated reagents may generate convincing data (Moore et al., 2022).

For cancer patients, immunotherapy has been considered an important and promising treatment, apart from surgery, chemotherapy, and radiotherapy, which has led to revolutionary changes in oncological treatment modalities (Li et al., 2021; Liu et al., 2022). However, the immunosuppressive tumor microenvironment (TME) is still a bottleneck that hampers immunotherapeutic efficacy (Lequeux et al., 2021). The TME is composed of distinct cell types, including immune cells, endothelial cells, mesenchymal stem cells, and fibroblasts. The reciprocal communication between infiltrating immune cells and tumor stem cells within the TME can facilitate tumor immune escape, recurrence and metastasis (Bayik and Lathia, 2021). With regards to immune cells in PTC, accumulating studies have revealed that Tregs, dendritic cells, and mast cells act as tumorigenesis facilitators, whereas CD8^+^ T cells and natural killer cells act as tumor suppressors within the TME (Galdiero et al., 2016; Varricchi et al., 2019; Ferrari et al., 2020). As PTC progresses, the abundance and proportion of cancer-facilitating immune cells, such as Tregs, neutrophils, and dendritic cells increased significantly, while those of antitumor immune cells, such as CD8^+^ T cells and natural killer cells relatively decreased (Xie et al., 2020). In our study, nine of the 21 types of immune cells had a noticeable relationship with differential immune cell infiltration, and CD8+T cells decreased significantly, while Tregs increased significantly in the high-risk group. More intriguingly, eight of the 21 types of immune cells demonstrated statistically significant differences in immune cell infiltration and CD8^+^ T cells levels decreased remarkably while the level of Tregs increased remarkably in PSG7 high-expression group. In addition, immune-related gene sets showed significant changes in both groups. Accordingly, it is reasonable to infer that PSG7 may play a pivotal role in PTC progression by altering the tumor immune microenvironment with increasing age. Indeed, numerous studies have confirmed that PSGs may participate in the adjustment of the innate immune system and immune tolerance during pregnancy (Snyder et al., 2001; Rayev et al., 2017; Zimmermann and Kammerer, 2021). In the future, elucidating the complex mechanism of how immune cells and tumor cells interact will offer a unique perspective for more precise and personalized immunotherapy (Kubli et al., 2021).

Our study has a few limitations. First, although we included as many samples with complete clinical information as possible for strict validation and standardized the downloaded data to improve comparability, we must never lose sight of the fact that sampling bias arising from tumor heterogeneity and multi-platform integration may only be reduced to a certain extent but not eliminated. Second, it may be interesting to examine the basic expression of these screened DEGs and hub genes in the development of PTC with western blot, quantitative real-time PCR, immunohistochemistry, immunofluorescence assays and so on. Although emerging evidence suggests a series of DEGs in PTC, such as CRCT1, IL37, and IGF2BP1, there are still no reliable candidates for therapeutic targets of PTC. It is necessary to identify more DEGs and to explore whether possible targeted genes affect the initiation and progression of PTC. Application of the molecular experiments, the identification of biomarkers in PTC may be feasible. Third, to clarify the functions of DEGs and hub genes in PTC, a clean loss-of -function and gain-on-function study with tissue-type specificity and cell-type specificity remain warranted. Signaling pathways are more diverse in PTC than originally thought, such as the interleukin 17 (IL-17) signaling pathway, the cytokine-cytokine receptor interaction pathway and WNT signaling pathway. Although several pathways have been identified, a series of molecular experiments may be useful to prove more detailed and strong proofs for the possible phenotype and pathway regulation of these screened genes underlying PTC. Finally, despite the fact that microarray-based bioinformatic analysis is a powerful tool in efficient understanding of molecular mechanisms and for identifying potential biomarkers underlying PTC, further experimental validations of PSG7 are needed at molecular, cellular, and organismal levels.

Altogether, the results of the combined bioinformatics analyses of PTC datasets from TCGA and GEO revealed that 146 DEGs, meaningful prognostic markers, altered key pathways, and reshaped tumor immune microenvironments were involved in PTC. Our findings suggest that PSG7 may be a promising age-related prognostic marker in PTC for the first time. Second, we explored the mechanism of PTC deterioration with increasing age using bioinformatics approaches, which resolved the long-standing enigma behind this phenomenon, although we know that age at diagnosis is a self-contained element of danger for PTC prognosis. Therefore, our findings provide valuable insights into the potential clinical efficacy of prognostic estimation and therapeutic guidance for the personalized management of PTC. This study opens a new door to understanding the underlying mechanism by which the prognosis of patients with PTC declines with age.

## Data Availability

The original contributions presented in the study are included in the article/Supplementary Material, further inquiries can be directed to the corresponding author.
