# Supplementary material for: PSG7 indicates that age at diagnosis is associated with papillary thyroid carcinoma: A study based on the cancer genome atlas data
Source: Front Genet. 2022 Oct 5;13:952981. doi: 10.3389/fgene.2022.952981 (PMC9579346; doi:10.3389/fgene.2022.952981)
Supplement: Supplementary file 1 [file DataSheet1.ZIP › Supplementary Material/S3.docx]

# Supplementary Tables

Table 1 Information for the data set of GSE29265

| **GEO Dataset** | **Platform** | **Control** | **Tumor** | **Type** | **Description** |
| --- | --- | --- | --- | --- | --- |
| GSE29265 | GPL570 | 20 | 20 | Expression profiling by array | [HG-U133_Plus_2] Affymetrix Human Genome U133 Plus 2.0 Array |

Table 2 Baseline data of TCGA-THCA

| Patients with PTC in TCGA | Number |
| --- | --- |
| Stage |  |
| I | 276 |
| II | 51 |
| III | 108 |
| IV | 51 |
| T stage |  |
| T1 | 141 |
| T2 | 160 |
| T3 | 163 |
| T4 | 22 |
| gender |  |
| male | 130 |
| female | 358 |

Table 3 GO BP enrichment analysis

| ID | Description | p.adjust |
| --- | --- | --- |
| GO:0030198 | extracellular matrix organization | 4.85E-11 |
| GO:0043062 | extracellular structure organization | 4.85E-11 |
| GO:0045229 | external encapsulating structure organization | 4.85E-11 |
| GO:0097530 | granulocyte migration | 8.86E-10 |
| GO:0071621 | granulocyte chemotaxis | 1.18E-09 |
| GO:0097529 | myeloid leukocyte migration | 3.30E-09 |
| GO:0030574 | collagen catabolic process | 1.94E-08 |
| GO:0060326 | cell chemotaxis | 2.07E-08 |
| GO:0019730 | antimicrobial humoral response | 2.44E-08 |
| GO:1990266 | neutrophil migration | 2.44E-08 |
| GO:0030595 | leukocyte chemotaxis | 2.91E-08 |
| GO:0030593 | neutrophil chemotaxis | 6.39E-08 |
| GO:0030199 | collagen fibril organization | 1.52E-07 |
| GO:1990868 | response to chemokine | 1.59E-07 |
| GO:1990869 | cellular response to chemokine | 1.59E-07 |
| GO:0070098 | chemokine-mediated signaling pathway | 2.75E-07 |
| GO:0032963 | collagen metabolic process | 4.31E-07 |
| GO:0050900 | leukocyte migration | 2.91E-06 |
| GO:0061844 | antimicrobial humoral immune response mediated by antimicrobial peptide | 3.74E-06 |
| GO:0002548 | monocyte chemotaxis | 6.51E-06 |

Table 4 GO MF enrichment analysis

| ID | Description | p.adjust |
| --- | --- | --- |
| GO:0048018 | receptor ligand activity | 6.20E-19 |
| GO:0030546 | signaling receptor activator activity | 6.99E-19 |
| GO:0005125 | cytokine activity | 9.88E-17 |
| GO:0005201 | extracellular matrix structural constituent | 4.29E-13 |
| GO:0001664 | G protein-coupled receptor binding | 1.34E-10 |
| GO:0005126 | cytokine receptor binding | 2.78E-10 |
| GO:0008009 | chemokine activity | 2.78E-09 |
| GO:0004222 | metalloendopeptidase activity | 1.19E-08 |
| GO:0030020 | extracellular matrix structural constituent conferring tensile strength | 4.32E-08 |
| GO:0042379 | chemokine receptor binding | 5.04E-08 |
| GO:0004175 | endopeptidase activity | 4.35E-07 |
| GO:0030414 | peptidase inhibitor activity | 2.25E-06 |
| GO:0008237 | metallopeptidase activity | 2.74E-06 |
| GO:0004252 | serine-type endopeptidase activity | 2.77E-06 |
| GO:0061135 | endopeptidase regulator activity | 3.13E-06 |
| GO:0005539 | glycosaminoglycan binding | 3.13E-06 |
| GO:0061134 | peptidase regulator activity | 3.13E-06 |
| GO:0004866 | endopeptidase inhibitor activity | 3.81E-06 |
| GO:0008236 | serine-type peptidase activity | 9.61E-06 |
| GO:0017171 | serine hydrolase activity | 1.28E-05 |

Table 5 GO CC enrichment analysis

| ID | Description | p.adjust |
| --- | --- | --- |
| GO:0005581 | collagen trimer | 1.30E-14 |
| GO:0062023 | collagen-containing extracellular matrix | 1.30E-14 |
| GO:0005788 | endoplasmic reticulum lumen | 7.60E-09 |
| GO:0005583 | fibrillar collagen trimer | 5.05E-07 |
| GO:0098643 | banded collagen fibril | 5.05E-07 |
| GO:0098644 | complex of collagen trimers | 5.03E-05 |
| GO:0001533 | cornified envelope | 0.00012967 |
| GO:0005796 | Golgi lumen | 0.00097235 |
| GO:0005859 | muscle myosin complex | 0.00143302 |
| GO:0016460 | myosin II complex | 0.00597542 |
| GO:0098992 | neuronal dense core vesicle | 0.00783483 |
| GO:0032982 | myosin filament | 0.00810022 |
| GO:0031045 | dense core granule | 0.02027512 |
| GO:0005584 | collagen type I trimer | 0.02027512 |
| GO:0005927 | muscle tendon junction | 0.02027512 |
| GO:0034774 | secretory granule lumen | 0.02338141 |
| GO:0031232 | extrinsic component of external side of plasma membrane | 0.02338141 |
| GO:0060205 | cytoplasmic vesicle lumen | 0.02338141 |
| GO:0042581 | specific granule | 0.02338141 |
| GO:0031983 | vesicle lumen | 0.02338141 |

Table 6 KEGG enrichment analysis

| ID | Description | p.adjust |
| --- | --- | --- |
| hsa04060 | Cytokine-cytokine receptor interaction | 5.72E-10 |
| hsa04061 | Viral protein interaction with cytokine and cytokine receptor | 3.33E-07 |
| hsa04974 | Protein digestion and absorption | 2.52E-06 |
| hsa04657 | IL-17 signaling pathway | 3.56E-06 |
| hsa04080 | Neuroactive ligand-receptor interaction | 4.27E-05 |
| hsa05146 | Amoebiasis | 4.47E-05 |
| hsa05323 | Rheumatoid arthritis | 0.00229273 |
| hsa04062 | Chemokine signaling pathway | 0.00370487 |
| hsa04512 | ECM-receptor interaction | 0.02433137 |
| hsa04614 | Renin-angiotensin system | 0.09245779 |
| hsa04726 | Serotonergic synapse | 0.12572218 |
| hsa04640 | Hematopoietic cell lineage | 0.15014883 |
| hsa04310 | Wnt signaling pathway | 0.15958228 |
| hsa04020 | Calcium signaling pathway | 0.35893928 |
| hsa04151 | PI3K-Akt signaling pathway | 0.41852637 |
| hsa00590 | Arachidonic acid metabolism | 0.45784219 |
| hsa00592 | alpha-Linolenic acid metabolism | 0.48077413 |
| hsa04911 | Insulin secretion | 0.49521685 |
